# A Systematic Review of Mesenchymal Epithelial Transition Factor (*MET*) and Its Impact in the Development and Treatment of Non-Small-Cell Lung Cancer

**DOI:** 10.3390/cancers15153827

**Published:** 2023-07-27

**Authors:** Embla Bodén, Fanny Sveréus, Franziska Olm, Sandra Lindstedt

**Affiliations:** 1 Department of Clinical Sciences, Lund University, 22184 Lund, Sweden; embla.boden_janson@med.lu.se (E.B.); fa4743sv-s@student.lu.se (F.S.); franziska.olm@med.lu.se (F.O.); 2 Wallenberg Center for Molecular Medicine, Lund University, 22184 Lund, Sweden; 3 Lund Stem Cell Center, Lund University, 22184 Lund, Sweden; 4 Department of Cardiothoracic Surgery and Transplantation, Skåne University Hospital, 22242 Lund, Sweden

**Keywords:** non-small cell lung cancer, mesenchymal epithelial transition factor, *MET*, targeted therapies, genetic alterations, biomarker, systematic review

## Abstract

**Simple Summary:**

Lung cancer is the type of cancer that kills the most people in the world each year. It is difficult to diagnose lung cancer in the early stages and there are only few treatment options available once the cancer has spread. The mesenchymal epithelial transition factor (*MET*) gene is of importance in lung cancer development, and mutations in this gene are related to poor prognosis. Consequently, it is important to develop new treatment options that specifically target the *MET* protein. In this systematic review, we aimed to summarize the existing knowledge on the impact of *MET* on lung cancer development and the effect of currently available medications. Our hope is that the findings of this systematic review will deepen the understanding of other researchers, possibly providing a guiding hand as to what may be most interesting to focus on in future research projects on this subject.

**Abstract:**

Lung cancer represents the leading cause of annual cancer-related deaths worldwide, accounting for 12.9%. The available treatment options for patients who experience disease progression remain limited. Targeted therapeutic approaches are promising but further understanding of the role of genetic alterations in tumorigenesis is imperative. The *MET* gene has garnered great interest in this regard. The aim of this systematic review was to analyze the findings from multiple studies to provide a comprehensive and unbiased summary of the evidence. A systematic search was conducted in the reputable scientific databases Embase and PubMed, leading to the inclusion of twenty-two articles, following the PRISMA guidelines, elucidating the biological role of *MET* in lung cancer and targeted therapies. The systematic review was registered in PROSPERO with registration ID: CRD42023437714. *MET* mutations were detected in 7.6–11.0% of cases while *MET* gene amplification was observed in 3.9–22.0%. Six studies showed favorable treatment outcomes utilizing *MET* inhibitors compared to standard treatment or placebo, with increases in PFS and OS ranging from 0.9 to 12.4 and 7.2 to 24.2 months, respectively, and one study reporting an increase in ORR by 17.3%. Furthermore, patients with a higher mutational burden may derive greater benefit from treatment with *MET* tyrosine kinase inhibitors (TKIs) than those with a lower mutational burden. Conversely, two studies reported no beneficial effect from adjunctive treatment with a *MET* targeted therapy. Given these findings, there is an urgent need to identify effective therapeutic strategies specifically targeting the *MET* gene in lung cancer patients.

## 1. Introduction

Lung cancer is one of the most common malignancies, causing 12.9% of cancer-related deaths worldwide, resulting in 1.3 million deaths annually [1,2,3,4]. Between 80.0 and 85.0% of lung cancer cases are non-small cell lung cancer (NSCLC) [3,5,6]. Late diagnosis is a major problem, contributing to the short median survival of approximately 18 months and the overall 5-year survival rate for lung cancer of 15–21%, depending on gender [7,8]. Up to 75.0% of patients with newly diagnosed NSCLC have locally advanced or metastasized disease at diagnosis, with a 5-year survival rate below 5.0% [1]. To this day, locally advanced or metastasized NSCLC is commonly treated with platinum-based chemotherapy, offering modest efficacy, with response rates of 20.0–30.0% and a plethora of side effects, or immunotherapy [3,5,9]. Targeted therapies for several types of cancer, including NSCLC, have emerged as a beneficial option for subsets of patients. Current treatment guidelines for advanced NSCLC call for broad molecular profiling to identify and guide the choice of potential targeted therapy options [10]. The proportion of patients with NSCLC receiving next generation sequencing (NGS) is low, which is in part due to insufficient availability of tumor tissue at the time of diagnosis. Approximately 60.0–65.0% of patients undergo testing for mutations in the epidermal growth factor receptor gene (*EGFR*) and less than 25.0% of patients are tested for alterations in the mesenchymal epithelial transition factor gene (*MET*) [11].

**Figure 1:**
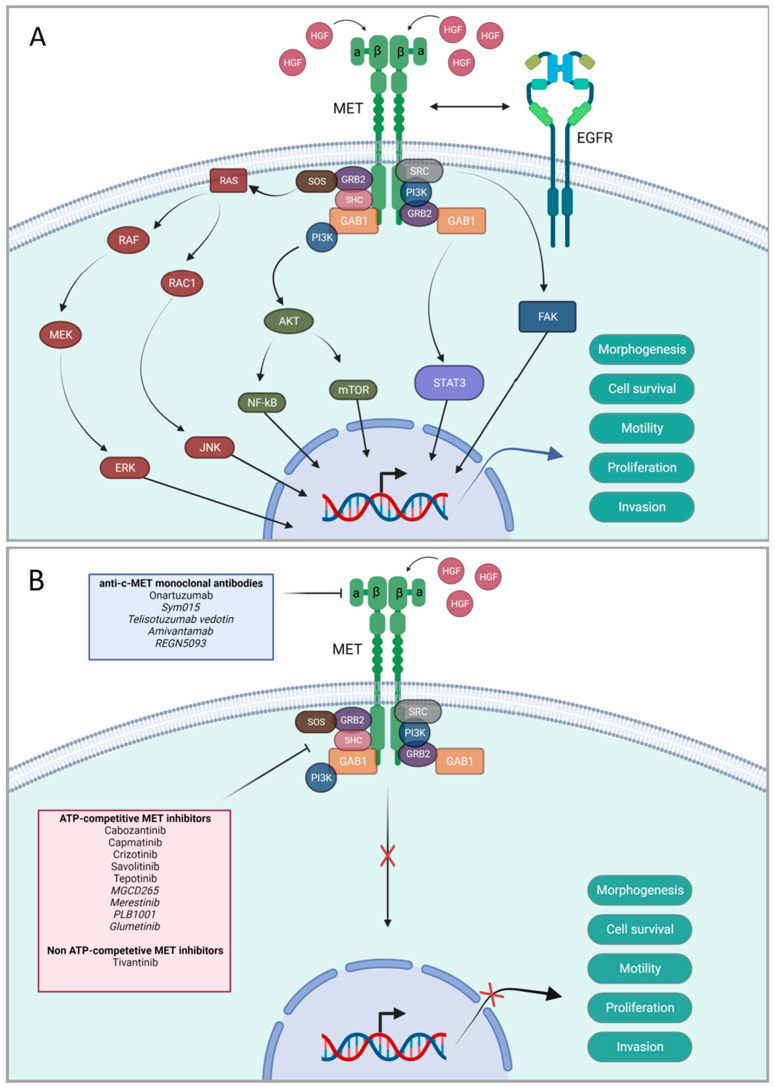
Schematic of the *MET* signaling pathway and the sites of action for *MET* targeted therapies. (**A**) The *MET* signaling pathway with downstream intracellular signaling and transcription of genes leading to enhanced morphogenesis, cell survival, motility, proliferation, and invasion. *MET* is activated by its ligand, HGF, and the *MET* receptor can interact in various ways with the *EGFR* receptor. (**B**) The *MET* targeted therapies presented according to specific targets, either extracellular or intracellular. The pipe-line drugs are highlighted in cursive. MET: mesenchymal epithelial transition factor, HGF: hepatocyte growth factor, EGFR: epidermal growth factor receptor, SRC: v-src sarcoma (Schmidt-Ruppin A-2) viral oncogene homolog, PI3K: phosphatidylinositol 3-kinase, GRB2: growth factor receptor-bound protein 2, GAB1: GRB2-associated binding protein 1, SHC: src homology 2 domain-containing, SOS: son of sevenless, RAS: rat sarcoma, RAF: rapidly accelerated fibrosarcoma, MEK: MAPK effector kinase, ERK: extracellular signal-regulated kinase, RAC1: ras-related C3 botulinum toxin substrate 1, JNK: janus kinase 1, AKT: ak strain transforming, NF-kB: nuclear factor kappa B, mTOR: mammalian target of rapamycin, FAK: focal adhesion kinase, STAT3: signal transducer and activator of transcription 3, ATP: adenosine triphosphate. Created in biorender.com.

*EGFR* is a transmembrane receptor that is involved in several signaling pathways; it promotes cell proliferation and is anti-apoptotic. Overexpression of the *EGFR* gene is a well-known pathological mechanism in NSCLC, present in 43.0–89.0% of NSCLC cases, which can lead to poorer outcomes [12]. Several *EGFR* TKIs exist; however acquired resistance to these therapies is common and alterations in the *MET* gene have been proven to be a contributing factor [13]. The protein *MET* is a transmembrane receptor tyrosine kinase (RTK) with a central role in cell motility, morphogenesis, proliferation, survival, and invasion (Figure 1A) [5,14,15,16]. Alterations in the *MET* gene, such as gene copy number (GCN) gain, mutation, or overexpression of the protein, have been reported in NSCLC [17]. The only known ligand to *MET* is hepatocyte growth factor (HGF) [6]. HGF is found in healthy lung tissue but is often overexpressed in NSCLC. Aberrant signaling through the HGF/*MET* pathway has clinically been linked to oncogenic potential and poor outcomes in NSCLC, with shortened overall survival (OS) and progression-free survival (PFS). However, studies showing the opposite also exist, introducing a contradiction in this area of research. One proven cause of unfavorable outcomes in *MET* altered lung cancer is acquired resistance to *EGFR* TKIs, underlining the need for more efficient targeted therapies [1,5,6,14,15,17,18,19,20]. The frequency of any form of dysregulation of *MET* in NSCLC ranges from 3.0 to 7.0%. Sporadic GCN gain of *MET* is detected in 1.0–4.0% of wild type *EGFR* NSCLC cases. *MET* exon 14 skipping mutations occur in approximately 3.0% of NSCLC cases [2,3,18,20]. Amplification of the *MET* gene is the most common type of dysregulated signaling in NSCLC with acquired resistance to *EGFR* TKIs, with reported frequencies between 5.0 and 26.0% [20,21]. Alterations in *MET* have been shown to upregulate the expression of *EGFR* ligands, which in turn increases *EGFR* signaling, promoting cell proliferation, angiogenesis, and apoptosis [15]. Increased expression of HGF can also promote resistance to *EGFR* TKIs by supporting clonal selection of a subpopulation of cells with *MET* amplification [1]. Despite the fact that acquired resistance to *EGFR* TKIs is very common, they remain the preferred first-line treatment for locally advanced or metastatic *EGFR* mutation-positive NSCLC [15,22,23]. For patients with acquired resistance to *EGFR* TKIs caused by upregulation or amplification of *MET*, it may be beneficial to treat with a combination of inhibitors of both *MET* and *EGFR*, as this has been shown to have a synergistic inhibitory effect on the proliferation of cancer cells [2,21,22]. A combination of *EGFR* and *MET* TKIs has been shown to possibly delay the occurrence of resistance to *EGFR* TKIs [24]. For an overview of the *EGFR* and *MET* TKIs discussed in this article, see Table 1 (Figure 1B).

An issue with these types of targeted therapies remains the inherent genetic heterogeneity of NSCLC. Several mutations may co-exist in the same patient and complicate the interpretation of specific drug effects [3,25]. An additional aspect to take into consideration is the variability in methodologies employed to quantify aberrant *MET* expression. Some studies use immunohistochemistry (IHC) with the definition of *MET* positivity ranging from 1+ to 3+ and *MET* negativity varying from 0 to 1+ [2,17,18,20]. Other studies apply gene copy number (GCN) ≥ 5 or *MET*/centromere 7 ratio ≥ 2.0 as the lower limit for defined *MET* positivity [20].

Genetic profiling carried out by NGS is a rapidly progressing area of research within oncology. The methods used are becoming increasingly cost and time efficient, allowing for improved individual genetic testing and tailored therapy options [26]. The aim of this systematic review was to provide a comprehensive and unbiased summary of the evidence investigating the role of *MET* and aberrant expression of the protein in lung cancer development and treatment thereof.

## 2. Methods

### 2.1. Search Strategy

**Figure 2:**
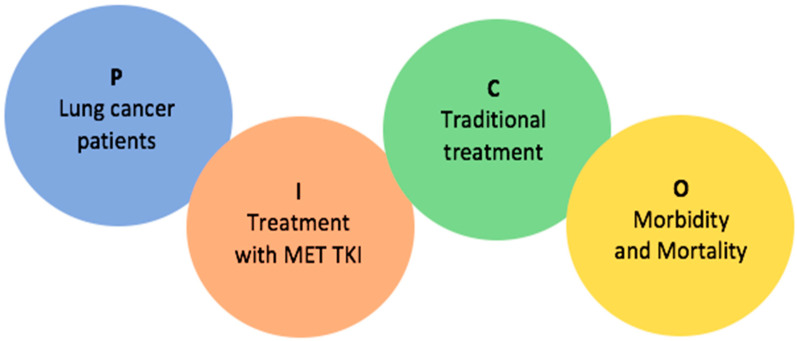
Illustration of the PICO model used to create the search queries used in this systematic review. P: population, I: intervention, C: comparison, O: outcome, *MET*: mesenchymal epithelial transition factor, TKI: tyrosine kinase inhibitor.

The Preferred Reporting Items for Systematic reviews and Meta-Analyses (PRISMA) guidelines were used in conducting this systematic review; for the full PRISMA 2020 Checklist, see Appendix B [27] (Table A1 and Table A2). A protocol was not established. The systematic review was registered in PROSPERO with registration ID CRD42023437714. Medically relevant databases containing existing literature were systematically searched by the authors for articles related to *MET*, lung cancer, and targeted *MET* therapies. The systematic search was conducted in Embase and PubMed in February of 2023 with the help of two search queries (Figure 2, Appendix A) based on the population, intervention, comparison, and outcome (PICO) model. Only clinical trials and randomized clinical trials were included.

### 2.2. Exclusion and Inclusion Criteria

**Figure 3:**
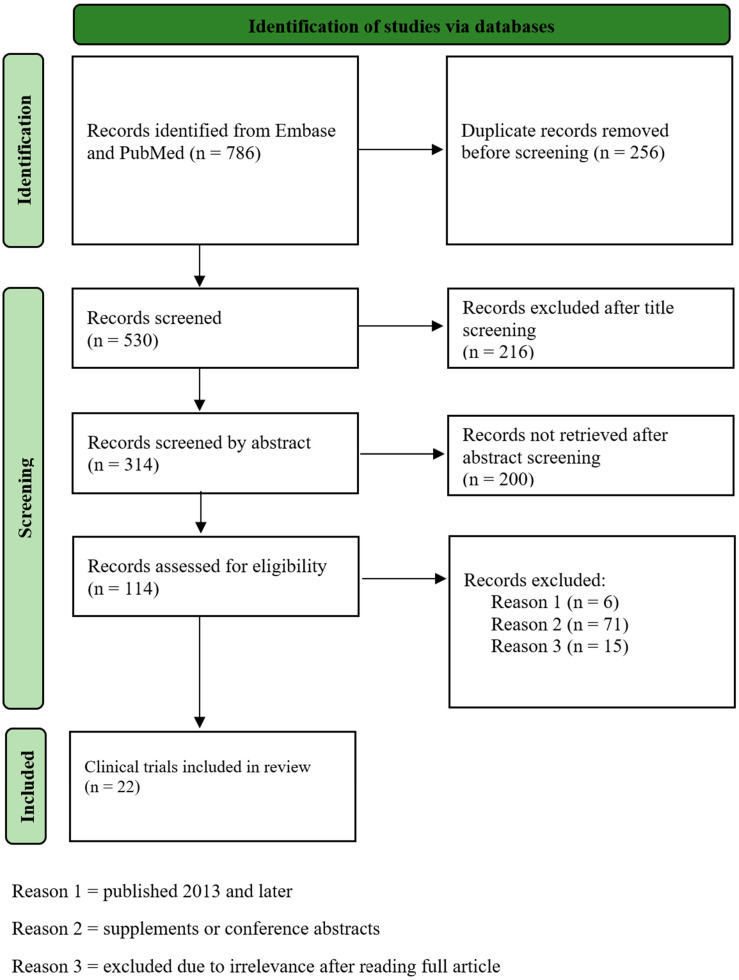
Flow diagram of inclusion and exclusion steps according to the PRISMA guidelines.

After the initial search in Embase and PubMed, all duplicates were removed. All remaining articles were screened independently by two of the authors, E.B. and F.S., for relevance based on the title, abstract, and full article. Articles were included if one or both parties deemed it relevant to the subject. Articles that were published in 2012 or earlier were excluded as well as supplemental materials and conference abstracts. A full text screening of all eligible articles was performed independently by the two parties, excluding irrelevant and ongoing trials. A flow diagram of exclusion steps is presented in Figure 3.

### 2.3. Data Extraction and Processing

The included papers were grouped by investigated drug; the main characteristics are described in Table 2 and Table 3. The main outcome measures presented and analyzed in this article are PFS and OS. Data on prevalence of *MET* mutations and amplifications in lung cancer were also analyzed and included in this article.

## 3. Bias

**Figure A1:**
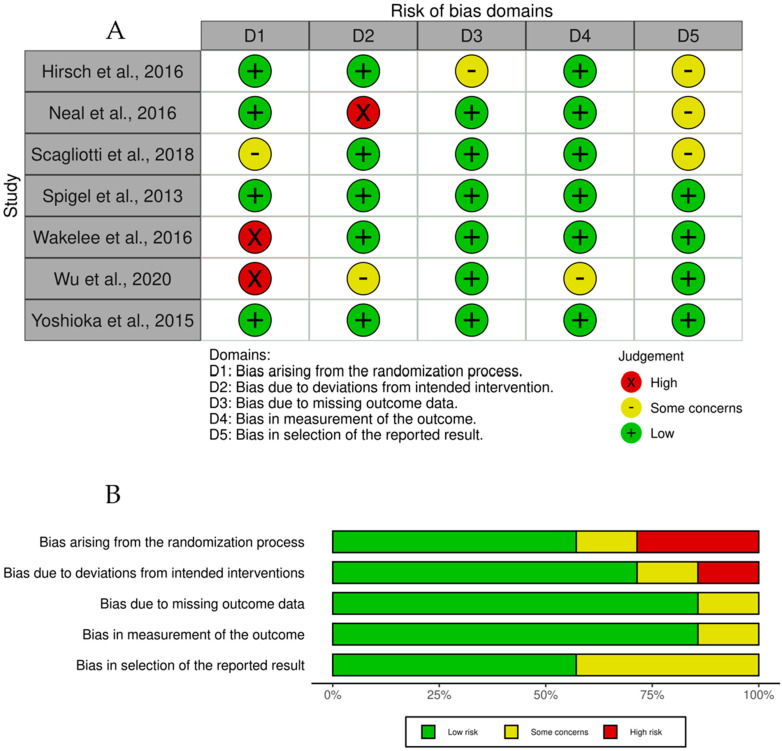
Risk of bias plots for all included RCTs in this systematic review, created in the Cochrane Risk of Bias Tool "robvis" [2,5,6,17,23,30,32,33]. (**A**) Traffic light plot, indicating the risk of bias for each included RCT in each of the five domains. (**B**) Summary plot, showing the summarized risk of bias in each domain, and an overall risk of bias measurement. D: domain, RCT: randomized controlled trial.

**Figure A2:**
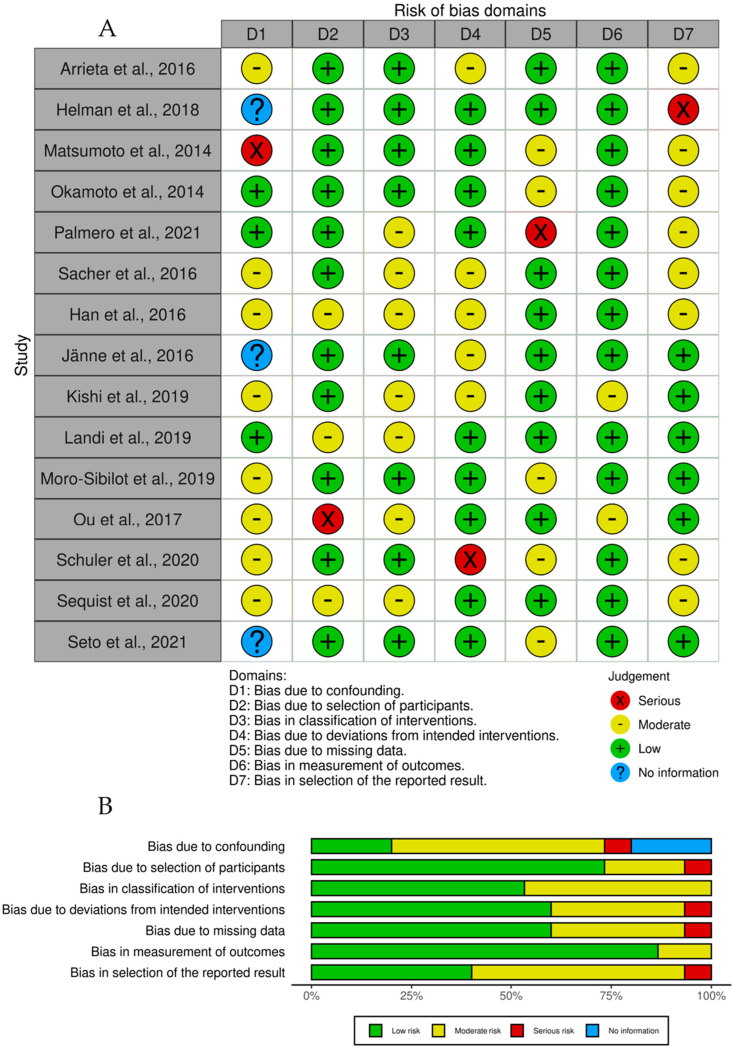
Risk of bias plots for all included clinical trials in this systematic review, created in the Cochrane Risk of Bias Tool "robvis" [1,3,11,14,15,18,19,20,21,22,24,25,28,29,31,33]. (**A**): Traffic light plot, indicating the risk of bias for each included clinical trial in each of the seven domains. (**B**): Summary plot, showing the summarized risk of bias in each domain, and an overall risk of bias measurement. D: domain.

Publication bias is a factor inevitably impacting systematic reviews due to positive selection bias in the publication of research articles. This may lead to overestimation of the therapeutic effect of the investigated drugs. In this systematic review, several articles reported no effect of the evaluated drugs on PFS and OS among patients with lung cancer. By simultaneous screening of all articles by separate parties, the risk of bias in the process of selecting articles to be included in this systematic review was minimized. Furthermore, the Cochrane Risk of Bias Tool “robvis” was adapted to create risk of bias plots for all RCT’s and clinical trials included in this systematic review, see Appendix C (Figure A1 and Figure A2) [33]. None of the authors of this systematic review declare any conflicts of interest related to the article.

## 4. Results

This systematic review identified a total of 786 eligible articles by the two search queries (Appendix A) employed following the PRISMA guidelines. Exclusion of 256 duplicates resulted in 530 remaining articles. After the assessment of article titles, 314 articles remained followed by further exclusion based on the abstract, resulting in 114 remaining articles. Six articles were excluded based on publication year and 71 studies were excluded due to being conference abstracts or supplements. Consequently, 37 full articles remained for the final full text screening. A total of 22 articles were judged to be relevant to this systematic review (Figure 3). Six of the included articles present data regarding the prevalence and significance of aberrant signaling through the HGF/*MET* pathway in lung cancer. The remaining 16 articles present data on the effect of specific targeted therapies on mortality and morbidity of lung cancer patients.

### 4.1. Epidemiology

The reported patient characteristics varied greatly between the included studies, see Appendix C (Table A3). Landi et al. reported the lowest mean age in all the studies (56.0 years) while Moro-Sibilot et al. reported the highest in their subgroup of patients with *MET* mutations (72.0 years) [18,29]. In regard to the included subjects’ gender, Matsumoto et al. included a vast overweight of women compared to men in their clinical trial (80.3% women), while Okamoto et al. reported the highest percentage of men included (77.0%) [3,19]. Similar to age and gender, smoking history also varied between the studies. The highest reported prevalence of never smokers was seen in the clinical trial conducted by Matsumoto et al., with 89.1% of the patients being never smokers in an *EGFR* WT population [19]. Interestingly, both of the *MET*-negative cohorts in a RCT by Spigel et al. held smaller fractions of patients with no smoking history (7.0% in the treated subgroup and 3.0% in the placebo subgroup) compared to the *MET*+ cohorts in the same study (20.0% in the treated subgroup and 19.0% in the placebo subgroup) [6]. In the study by Moro-Sibilot et al., the authors reported double the amount of patients with a no smoking history in the *MET* mutated cohort compared to the *MET* amplified cohort (*MET* mutated: 48.0%, *MET* amplified: 24.0%) (Table A3) [29].

### 4.2. Prevalence of Aberrant MET Expression

The reported prevalence of the different *MET* gene alterations varies greatly between the articles included in this systematic review. In a prospective clinical trial by Palmero et al., genetic testing of treatment naïve NSCLC patients was carried out to identify genetic alterations. Next generation sequencing (NGS) of circulating cell free tumor DNA in blood was compared to standard-of-care tissue-based biopsy testing in order to determine the patients’ mutational burden. The results showed no difference between the methods’ ability to identify mutations in NSCLC. Out of 186 tested patients, 11.0% harbored *MET* exon 14 skipping mutations (*MET*ex14) and 22.0% harbored amplification of the *MET* gene [11].

In a retrospective clinical study by Okamoto et al., 295 patients were tested for *MET* mutations and 229 patients were tested for *MET* amplification. The results showed a prevalence of 7.6% for mutations in the *MET* gene and 3.9% for *MET* amplification, with a median GCN of 8.8. The median OS was found to be non-significantly longer among patients without *MET* amplification [3].

Sacher et al. carried out a single arm, single center clinical trial, testing the *EGFR* TKI erlotinib on 22 NSCLC patients with varying *EGFR* mutational statuses. All included patients were naïve to any systemic anti-cancer treatment. *MET* amplification was determined by fluorescence in situ hybridization (FISH) and *MET* positivity was defined as GCN ≥ 4. Protein expression was assessed through IHC and *MET* positivity was defined as an H-score ≥ 100. The prevalence of any aberrant *MET* expression in this study was 45.0%, while *MET* amplification specifically was seen in 9.0% of the patients [28].

Helman et al. conducted a retrospective study that performed NGS on plasma samples from patients suffering from NSCLC harboring *EGFR* mutations. NGS was carried out at study enrolment before receiving treatment and after disease progression to evaluate the effect of the *EGFR* tyrosine kinase inhibitor (TKI) rociletinib. Prior to treatment with rociletinib, 15.0% of the NGS screened patients were positive for genetic alterations in the *MET* gene. After disease progression on treatment with rociletinib, another 7.6% of the patients with acquired resistance to the drug had acquired amplification of the *MET* gene. This amplification was found to be caused by focal amplification in 4.5% of the patients and by aneuploidy in the remaining 3.1% [25].

In a phase II study by Arrieta et al., the levels of the HGF protein were analyzed in 66 patients with either *EGFR* mutated or *EGFR* wild type lung adenocarcinoma. All patients received treatment with the *EGFR* TKI afatinib after disease progression on first-line platinum-based chemotherapy. Patients with reduction of HGF levels after treatment with afatinib had a significantly longer PFS, OS, and objective response rate (ORR) compared to patients with higher levels of HGF after treatment with afatinib. These differences were most prominent among patients with *EGFR* mutated disease. The authors of the study suggest that HGF has a direct role in acquired resistance to *EGFR* TKIs, making HGF an interesting target in this field of research [1].

In a different phase II study by Matsumoto et al., HGF levels were measured and *MET* mutational status was determined in 47 patients with *EGFR* wild type NSCLC treated with erlotinib. Tumors expressing HGF had a poor response to erlotinib, and the patients had a shorter median PFS compared to the HGF negative study population. *MET* mutational status did not impact PFS or the response to erlotinib in this clinical trial [19].

### 4.3. Targeted Therapies

Sixteen of the papers included in this systematic review are studies investigating targeted therapies for the *MET* gene. The different *MET* TKIs evaluated in the clinical trials are presented in Table 3. The most common *MET* TKIs were onartuzumab and crizotinib, occurring in five and four of the included articles each.

In a phase II study conducted by Hirsch et al., treatment with the combination of onartuzumab, paclitaxel, and carboplatin/cisplatin was compared to treatment with only paclitaxel and carboplatin/cisplatin in 106 NSCLC patients. The included patients were all *EGFR* wild type, with or without mutations in the *MET* gene. The results showed no significant benefit of added treatment with onartuzumab, regardless of *MET* mutational status, with similar PFS (5 months in the onartuzumab group and 5.2 in the placebo group) and OS (10.8 months vs. 7.9 months) in the two groups. In this study, *MET* mutational status was evaluated by immunohistochemistry, with IHC3+ and IHC2+ considered *MET* positive, and IHC1+ and IHC0 considered *MET* negative [17].

Similar results were found in a phase II study conducted by Wakelee et al., including 259 NSCLC patients in two cohorts. Cohort 1 tested the addition of onartuzumab vs. placebo to treatment with bevacizumab, carboplatin/cisplatin, and paclitaxel. In cohort 2, patients received either onartuzumab or placebo in addition to carboplatin/cisplatin and pemetrexed. The subjects had varying mutational statuses regarding both *EGFR* and *MET*. *MET* positivity was defined as IHC3+ or IHC2+. In cohort 1, the overall median PFS was 5.0 months in the onartuzumab group compared to 6.8 months in the placebo group. In a *MET*+ subgroup, median PFS was 4.8 months and median OS was 9.9 months in the onartuzumab arm compared to 6.9 months and 16.5 months in the placebo arm. Cohort 2 revealed similar results, with a median PFS of 5.1 months in the placebo group compared to 4.9 months in the onartuzumab group. Median OS was 13.7 months in the placebo treated group and 8.5 months in the onartuzumab treated group. In the *MET*+ subgroup of cohort 2, median PFS was 5.0 in for both onartuzumab and placebo while median OS was marginally longer in the onartuzumab arm (8.0 months) compared to the placebo arm (7.6 months) [32].

In another phase III study evaluating onartuzumab, 636 NSCLC patients with varying *EGFR* and *MET* mutational statuses were included and treated with either erlotinib and onartuzumab or erlotinib and placebo. In this study, high doses of onartuzumab were associated with a longer median PFS compared to lower doses of erlotinib or placebo (high dose PFS = 4.37; low dose PFS = 2.5 months; placebo PFS = 2.5 months). No significant differences were found in OS regardless of *MET* mutational status [14].

In contrast to this, a phase II study by Spigel et al. performed on NSCLC patients found that both median PFS and OS were significantly longer in a *MET* positive subgroup (defined as *MET* IHC3+ or IHC2+) treated with onartuzumab and erlotinib compared to a subgroup treated with erlotinib and placebo (dual treatment PFS = 2.9 months, OS = 12.6 months; single treatment PFS = 1.5 months, OS = 3.8 months). All 136 included participants were *EGFR* wild type but had varying *MET* mutational statuses. Interestingly, the patients without any mutations in the *MET* gene had earlier progression when treated with the combination of onartuzumab and erlotinib compared to erlotinib and placebo [6].

A third phase II study, titled the global METLung study (OAM4971g), presented results on treatment with onartuzumab and erlotinib without comparing the effects to a control group. This study included 61 patients with *MET* and *EGFR* mutation positive NSCLC but was terminated early due to lack of efficacy of onartuzumab. Patients with *MET* IHC3+ or IHC2+ as well as a total number of *MET* genes in 20 cancer cells ≥ 90 as determined by a gene amplification assay were considered *MET* positive. The preliminary results showed a median PFS of 8.5 months, a median OS of 15.6 months, and an overall response rate of 68.9% in the patients treated with onartuzumab and erlotinib [15].

In a phase II clinical trial, Landi et al. evaluated treatment with the targeted *MET* inhibitor crizotinib in an *EGFR* wild type NSCLC population of 26 patients with either *MET* amplification or *MET*ex14 mutation. The median PFS was 4.4 months, median OS was 5.4 months, and the ORR was 27.0%. *MET* amplification was defined as a *MET*/centromere 7 (CEP7) ratio > 2.2 [18]. Jänne et al. treated 67 NSCLC patients with mixed *EGFR* mutational statuses with crizotinib and dacomitinib in a phase I clinical trial. The included patients had experienced progression on first-line treatment with either chemotherapy or another targeted therapy and were included in either an escalation phase cohort or in an expansion phase cohort. The median PFS was 3.0 months in the escalation cohort, with 61.0% of these patients having stable disease during the treatment. In the expansion cohort, the median PFS was 2.1 months and 32.0% of the patients had stable disease during treatment. No association was seen between overexpression of *MET* and PFS. *MET* positivity was defined as IHC3+, IHC2+, or *MET* GCN ≥ 2.1 [21].

In another phase II clinical trial conducted by Moro-Sibilot et al., 53 NSCLC patients with varying *EGFR* mutational statuses were treated with crizotinib. The study included two cohorts with aberrant *MET* expression, one with *MET* GCN ≥ 6 (*n* = 25) and one with positivity for *MET* exon skipping mutations in exon 14 or 16–19 (*n* = 28). In the *MET* amplified cohort (GCN ≥ 6), the ORR was 16.0%, the median PFS was 3.2 months, and the median OS was 7.7 months. In the *MET* mutated cohort, the OS was longer while the PFS and ORR were inferior (ORR = 10.7%, median PFS = 2.4 months, median OS = 8.1 months) [29].

In a phase I study by Ou et al., crizotinib was evaluated in combination with erlotinib on 26 NSCLC patients with previous progression of disease on one or two prior treatments with chemotherapy. The patients’ *EGFR* and *MET* mutational statuses were not reported in this article. Only 20 of the 26 included patients were evaluated for response to treatment. Of these, two had partial response, eight had stable disease, and ten had progressive disease as defined by the RECIST version 1.1 guidelines [24].

One study evaluated the *MET* targeted drug savolitinib in combination with osimertinib in a phase Ib clinical trial. A total of 180 patients with *EGFR* mutation positive and *MET* amplified NSCLC received treatment with savolitinib and osimertinib after previous treatment with one, two, or three different *EGFR* TKIs. In this study, *MET* amplification was defined as *MET* GCN ≥ 5, *MET*/CEP7 ratio ≥ 2, *MET* IHC3+, or *MET* expression in ≥20.0% of tumor cells as determined by NGS. Patients were stratified into one of two cohorts according to type of *EGFR* mutation and number of prior *EGFR* TKI treatments. Only 161 patients were eligible for final evaluation of treatment effect. Of these, 89 patients had a partial response according to the RECIST version 1.1 guidelines. One of the two cohorts included 138 patients and had a median PFS of 7.6 months, while the other cohort, including 23 patients, had a median PFS of 9.1 months [22].

In a phase Ib/II trial by Wu et al., standard platinum doublet chemotherapy was compared to treatment with the targeted drugs tepotinib and gefitinib. In this clinical trial, 55 patients were included, all with NSCLC, positive for *EGFR* mutation, and *MET* overexpression or amplification. *MET* overexpression was defined as IHC2+ or IHC3+, and amplification was defined as GCN ≥ 5. All patients included in the phase II part of the trial had acquired resistance to other first or second-generation *EGFR* TKIs. There were no significant differences in median PFS or OS when comparing chemotherapy to targeted therapy. Subgroup analyses were carried out on groups of patients harboring either *MET* amplification or high *MET* overexpression defined as IHC3+. In the *MET* amplified group, both PFS and OS were significantly longer in the targeted therapy treated group compared to the chemotherapy treated group (targeted therapy median PFS = 16.6, chemotherapy median PFS = 4.2; targeted therapy OS = 37.7, chemotherapy OS = 13.1). In the *MET* IHC3+ group, the median PFS was 8.3 months, and the OS was 37.3 months compared to a PFS of 4.4 months and an OS of 17.9 in the chemotherapy group [23].

A phase I study evaluating capmatinib in NSCLC patients with *MET* amplification or *MET* overexpression showed that patients with a high *MET* GCN or *MET*ex14 mutations may benefit from treatment with *MET* inhibitors. Of 55 enrolled patients treated with capmatinib, 26 were included in a dose expansion group with varying *EGFR* mutational statuses and either *MET* overexpression or amplification. The remaining 29 patients were all *EGFR* wild type and had high overexpression of *MET*, defined as IHC3+. Complete response was observed in one of the 55 patients and partial response was seen in ten patients. The median PFS was 3.7 months for the entire cohort, whereas the *MET* IHC3+ group had a median PFS of 5.1 months. An even greater PFS was seen among patients with *MET* GCN ≥ 6 (median PFS of 9.3 months). Furthermore, in four patients with *MET*ex14 mutations, a reduction in the tumor burden between 14.0 and 83.0% could be seen. This trial was terminated early due to disease progression and frequent adverse events [20].

A phase II trial by Seto et al. tested the effect of capmatinib on 45 *EGFR* wild type NSCLC patients divided into several cohorts depending on *MET* mutational burden and prior systemic anti-cancer treatment. Aberrant *MET* expression was defined as the presence of the *MET*ex14 mutation or amplification of the *MET* gene. The results showed that treatment with capmatinib as a second or third-line option in *MET*ex14 positive subjects (*n* = 11) yielded an overall response rate of 36.4%. The overall response rate in a cohort of *MET* amplified patients with GCN ≥ 10 (*n* = 11) was found to be 45.5%. In contrast to this, in a cohort with *MET* GCN ≥ 4 but <6 (*n* = 10), the overall response rate was only 10.0%. The remainder of the patients were further subdivided into considerably smaller cohorts with inconclusive results [31].

Yoshioka et al. conducted a phase III clinical trial comparing treatment with tivantinib and erlotinib to treatment with erlotinib and placebo in 303 *EGFR* wild type NSCLC patients. All included patients had received one or two prior treatments, one of them being platinum-based chemotherapy. The patients had varying *MET* mutational statuses, including high and low expression of *MET* as well as elevated and normal *MET* GCN. The authors found no significant difference in the median OS when comparing the dual treatment group to the erlotinib plus placebo group. In the patients treated with both tivantinib and erlotinib, a significantly longer median PFS could be seen compared to the placebo group (dual treatment PFS = 2.9 months, placebo PFS = 2.0 months). Furthermore, high expression of HGF, defined as an H-score ≥ 200 as measured by IHC, was associated with a significant benefit in OS in the tivantinib plus erlotinib group compared to the placebo group. This clinical trial was terminated early due to increased incidence of interstitial lung disease, with 14 cases in the dual treatment group and 6 cases in the placebo group [5].

This is coherent with the results of a phase III study conducted by Scagliotti et al., which also compared erlotinib plus tivantinib to erlotinib plus placebo in 109 *EGFR* mutated NSCLC patients with varying *MET* mutational statuses. Here the median PFS was 13.0 months and the median OS was 25.5 months in the dual treatment group compared to a median PFS of 7.5 months and a median OS of 20.3 months in the erlotinib plus placebo group [30].

In a phase II clinical trial, Neal et al. assessed treatment with cabozantinib in *EGFR* wild type NSCLC patients with varying *MET* mutational statuses. This study comprised three treatment arms, one with both erlotinib and cabozantinib, one with only cabozantinib, and one with only erlotinib. All patients had previously been treated with one or two other therapeutic agents prior to enrolment in this trial. The median PFS was significantly longer in the erlotinib plus cabozantinib arm (median PFS = 4.7 months) and in the cabozantinib arm (median PFS = 4.3 months) compared to the erlotinib arm (median PFS = 1.8 months). The median OS was also longer in the erlotinib plus cabozantinib arm (median OS = 13.3 months) compared to the erlotinib arm (median OS = 5.1 months). The median OS in the cabozantinib arm was 9.2 months and was not significantly longer compared to the erlotinib arm. In this clinical trial, no association was found between *MET* IHC status and median PFS in the patient groups receiving treatment with cabozantinib or erlotinib plus cabozantinib [2].

**Figure A3:**
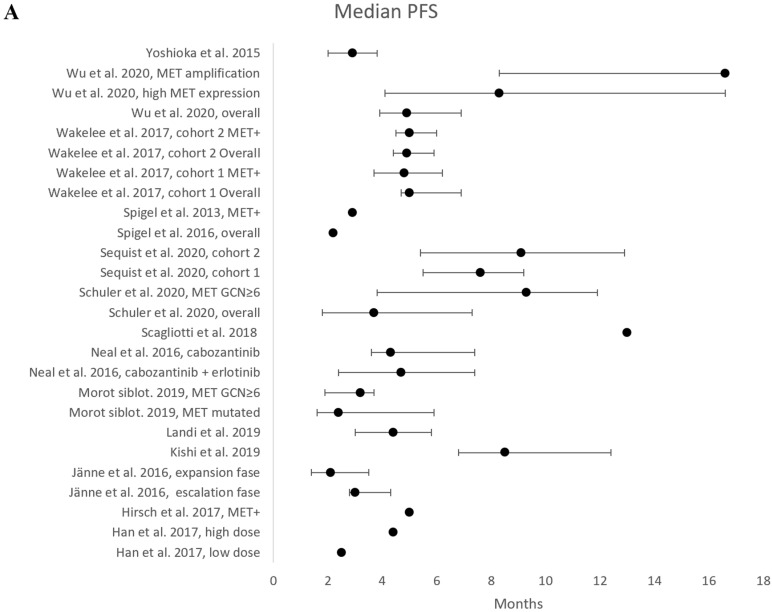

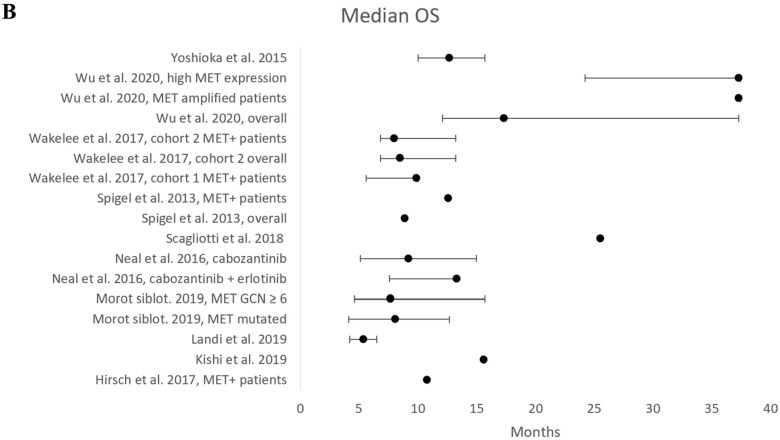
Forest plots presenting the median PFS and median OS with 95% confidence intervals (CI) from the included articles in this systematic review presenting results on treatment with a MET TKI. Results that are specific to a MET+ subgroup are presented separately from the overall cohort results [2,5,6,14,15,17,18,20,21,22,23,29,30,32]. (**A**) Median PFS with 95% CIs. (**B**) Median OS with 95% CIs.

In summary, many of the targeted drugs mentioned in this review show promising results that encourage further research. Onartuzumab, as an added treatment to erlotinib, has been shown in several trials to lead to a longer PFS and OS in NSCLC patients, compared to treatment with placebo and erlotinib. One clinical trial showed a reversed effect of onartuzumab as an added treatment to chemotherapy, as compared to placebo, with a shortened PFS and OS among the patients treated with onartuzumab. Crizotinib alone has been proven to greater increase OS among NSCLC patients harboring *MET* mutations compared to patients with *MET* amplified cancer genotypes. Crizotinib, as an addition to the *EGFR* TKIs dacomitinib or erlotinib, has resulted in a greater fraction of patients with stable disease. Treatment with savolitinib in combination with osimertinib has led to a fraction of patients with partial response of more than 55% in cohorts of NSCLC patients with previous progression of disease on *EGFR* TKIs. Tepotinib and gefitinib, as a treatment for NSCLC patients harboring high overexpression or amplification of *MET*, has been shown to prolong both the PFS and OS compared to treatment with chemotherapy. Similarly, in NSCLC patients with a high *MET* GCN or high levels of overexpression of *MET*, treatment with capmatinib has prolonged PFS and led to a reduced tumor burden and higher overall response rates compared to cohorts with lower grades of overexpression or amplification. The addition of tivatinib to treatment with erlotinib has been proven to be effective and has led to increases in PFS and OS compared to placebo. Lastly, use of cabozantinib alone or in combination with erlotinib has led to significantly longer PFS compared to treatment with erlotinib alone. For an overview of the results, see Figure A3 (Appendix C).

## 5. Discussion

Lung cancer continues to be the leading cause of global annual cancer-related mortality, and the need for more efficient therapies is evident. Targeted therapies are a promising area of research, offering new possibilities for inhibiting genetic alterations that are involved in driving tumorigenesis, but to this day, there are no known clinically useful biomarkers [34,35,36,37]. Lung cancer patients harboring alterations in the *MET* gene detected in circulating DNA in blood or tumor tissue have been shown to have poorer outcomes.

In a study by Andreasson et al., the authors were able to show that the *MET* protein could be found in exhaled breath as well as in blood plasma, and that the expression of the protein diminished after surgical removal of the lung cancer, underlining the proteins role in lung cancer tumorigenesis [34].

There is a pressing need for novel and more efficient therapies that can target the *MET* signaling pathway, but for this to be possible, the understanding of how abundant certain genetic alterations are, and how they affect and drive lung cancer development, needs to be further deepened. Therefore, this systematic review aimed to summarize and enlighten the current status of genetic mapping and prevalence of different alterations in the *MET* gene in NSCLC patients, as well as existing and pipe-line targeted therapies towards this protein.

The articles reviewed in this paper reported that *MET* mutations were found in 7.6–11.0% of lung cancer patients and that amplification was found in 3.9–22.0% of cases [3,11]. One study reported that genetic alterations in the *MET* gene were present in 15.0% of the lung cancer cases analyzed [25]. These numbers are in line with previously published data [38,39,40]. One study presented results on prevalence, which differed significantly from the rest of the included papers, with a reported frequency of aberrant *MET* expression of 45.0% [28]. The reported prevalence of aberrant *MET* expression varies in different studies. This may, in part, be due to the fact that different studies analyze samples from different genotypes of lung cancer, with differing characteristics, using different quantification methods and definitions. Some of the articles included in this systematic review analyzed the prevalence of aberrant *MET* signaling in *EGFR* mutated patients, while other studies do not specify *EGFR* mutational status [3,11,25]. It has previously been shown that alterations in the *MET* gene are a known mechanism of resistance to *EGFR* TKIs; therefore, considering the impact of previous treatments on genetic analyses results may offer valuable insights for included patients [22].

The varying patient characteristics in the included studies make it difficult to predict any specific groups of the population that are at increased risk of developing NSCLC with *MET* mutated genotypes. Some trials do not specify *MET* mutational status or present their data on patient characteristics in cohorts of patients with varying *MET* mutational statuses [6,21,24]. However, some trends can be seen among the studies presenting results on patient characteristics in cohorts with specified *MET* mutations [6,15,18,20,22,23,29,31]. The median age among patients harboring *MET* mutations ranges from 56.0 to 72.0 years. *MET* mutations appear equally among both genders, with the greatest difference being reported by a single study being 32% men and 68% women [29]. Cohorts with *MET* positive patients reported smoking history as “never smokers” ranging from 19 to 72%, compared to cohorts consisting of *MET* negative patients, with the lowest reported percentage of never smokers being 3.0% in the study by Spigel et al. [6]. This could be indicative of a trend with higher fractions of *MET* positive NSCLC patients being never smokers. However, more research that is more directly aimed at this research question needs to be conducted to verify these results.

Five of the included studies evaluated the *MET* TKI onartuzumab. Spigel et al. found a significantly longer OS and PFS in *MET* positive patients receiving treatment with both onartuzumab and erlotinib compared to patients receiving erlotinib alone. Interestingly, they also found that the *MET* negative patients suffered earlier progression of disease and increased mortality when treatment with onartuzumab was added compared to treatment with placebo alone [6]. This is in line with the findings of Han et al., who showed a significantly longer PFS among patients treated with high doses of onartuzumab together with erlotinib compared to single treatment with erlotinib or erlotinib plus lower doses of onartuzumab [14]. Kishi et al. presented similar results regarding OS to what Spigel et al. presented; however, there was a remarkable difference in PFS between the two studies (Kishi et al. PFS = 8.5 months, Spigel et al. PFS = 2.9 months) [6,15]. Hirsch et al. and Wakelee et al. were not able to show a longer OS or PFS with the addition of onartuzumab to standard chemotherapy in lung cancer patients with varying *MET* mutational statuses [17]. *MET* has previously been suggested to act as both a suppressor and an oncogene, which is in line with the presented results [6]. These findings underline the importance of genetic testing upon lung cancer diagnosis in order to customize targeted therapies and postpone or prevent progression caused by treatment with ill-suited therapies.

Two separate trials investigated the effect of dual treatment with tivantinib and erlotinib compared to single treatment with erlotinib. They both found a prolongation in the median PFS in the dual treatment group compared to treatment with erlotinib alone, although to very differing extents. No difference was seen in the median OS [5,30]. One study included only *EGFR* wild type subjects while the other had only *EGFR* mutated subjects. The variation in *EGFR* mutational status is likely a contributing factor to the varying results of the studies included in this review. Some clinical trials included only *EGFR* wild type lung cancers, others included only *EGFR* positive cases or a combination of wild type and positive cases. Two studies evaluated the effect of additional treatment with onartuzumab to erlotinib in patients with varying *EGFR* mutational statuses [6,14]. As erlotinib is an *EGFR* TKI, it seems likely that the patients with an *EGFR* positive cancer genotype would benefit more from treatment with erlotinib than the patients with an *EGFR* wild type genotype, making it difficult to evaluate the actual effect of the added onartuzumab or capmatinib treatment. Therefore, in these studies, the effect of added treatment with onartuzumab or capmatinib should be interpreted carefully and further research in larger clinical trials is needed to determine the effect.

Multiple studies performed on different *MET* TKIs have presented varying results on PFS, ranging from 2.1 to 9.1 months, without comparisons to control groups [18,20,21,22,29]. Interestingly, two of these articles showed that treatment with the targeted drug crizotinib had a greater effect in a *ROS1* translocated NSCLC cohort compared to *MET* altered cohorts [18,29]. Other studies evaluating crizotinib did not report *ROS1* mutational status, rendering the results more difficult to interpret [21,24]. Neal et al. reported that treatment with cabozantinib and erlotinib and single treatment with cabozantinib was superior to treatment with erlotinib alone in lung cancer patients with *EGFR* wild type lung cancer. These results support the statement that patients with *EGFR* wild type lung cancer may not respond to treatment with *EGFR* TKIs alone. In the clinical trial conducted by Neal et al., the authors did not find any association between *MET* IHC+ status and prolonged PFS in the treatment groups receiving cabozantinib. It is not possible to draw any conclusions regarding the association between prolonged time to progression and death, and inhibition of the *MET* receptor specifically, as cabozantinib has multiple gene targets [2,22]. Several clinical trials present similar results, indicating that patients with a high mutational burden in the *MET* gene (defined as high overexpression, high GCN gain, and high IHC+ status) benefit more from treatment with *MET* TKIs compared to patients with a lower mutational burden [5,20,23,31]. This further emphasizes the importance of thorough genetic mapping of cancer patients prior to treatment.

An important aspect in trials testing novel drugs is the fact that the included cohorts differ greatly in cancer genotype [6,14]. This puts the reliability and comparability of the results into question. Another aspect is the fact that the drugs presented in these trials typically show a gain in PFS and OS months at best [5,6,14,17]. This needs to be put in relation with the potential side effects and adverse events that the patients experience during treatment [5,20]. *MET* TKIs are still considered experimental, and treatment with targeted *MET* inhibitors is currently a second, third, or even fourth-line treatment option. It is known that cancers generally harbor fewer driver mutations in the beginning of tumorigenesis compared to the later stages of disease and so it is possible that earlier implementation of *MET* inhibitors could provide a greater effect on PFS and OS [41,42]. However, the timing of treatment will need to be investigated in further clinical trials.

The different cancer genotypes also contribute to potential bias in reporting more or less promising pipe-line drugs. However, there are several interesting ongoing clinical trials evaluating different pipe-line drugs on more targeted patient groups harboring different *MET* alterations (Table 4) [43]. Two of these ongoing trials have already presented results from their studies. The NCT02544633 trial tested the *MET* inhibitor MGCD265 on patients with NSCLC and *MET* activating mutations or *MET* amplifications. The patients were divided into four study arms, one with *MET* activating mutations in tumor tissue, one with *MET* activating mutations in circulating tumor DNA in the bloodstream, one with *MET* amplification in tumor tissue, and one with *MET* amplification in circulating tumor DNA. Interestingly, in the case of *MET* amplification, the patients with *MET* amplification identified in circulating tumor DNA had a poorer PFS (2.76 months) and OS (4.08 months) compared to the patients with *MET* amplification identified in tumor tissue (PFS 4.85 months, OS 7.04 months). The patients with *MET* activating mutations in tumor tissue had a longer OS of 16.32 months compared to all other study arms, but not PFS (3.95 months). The last treatment group with *MET* activating mutations in circulating tumor DNA had a similar PFS to the other three study arms (3.39 months). No results were available regarding OS in this fourth study arm [44]. This data is similar to results from earlier studies on *MET* TKIs presented in this systematic review. A possible explanation to why the patients with *MET* amplification detected in circulating tumor DNA had a poorer outcome is the greater tumor burden associated with circulating tumor cells compared to that of a localized tumor.

One other study had available preliminary results at the time of writing this systematic review (trial ID: NCT02648724). In this phase I/II study, the monoclonal anti *MET* antibody mixture entitled Sym015 was investigated. The study included 57 patients divided into three different treatment arms, one with KRAS wild type patients with *MET* amplifications, one with *MET* amplified NSCLC, and one where the patients harbored *MET*ex14 deletions. The outcome measure presented was the ORR and the results showed that the KRAS wild type cohort had 0.0% ORR while the *MET* amplified and the *MET*ex14 deletion cohort both had an ORR of 25.0% [45]. A vast difference between the already existing and published clinical trials and the ongoing ones is the greatly improved consistence in genotypes included in the newer trials. The majority of the current trials on pipe-line targeted therapies consist of cohorts of patients that are all genetically altered in the *MET* gene rather than cohorts of mixed cancer genotypes. This will make future results more easily interpreted and clinically useful.

## 6. Strengths and Limitations

The most apparent limitation of the articles included in this review is the lack of control groups in most of the phase I trials, leading to difficulties in interpreting the results. Furthermore, some articles have a low number of included patients and overall, the number of participants varies greatly. Since the included studies have a high grade of variation in patient characteristics, such as age, gender, and smoking history, the comparison between the studies’ results need to be interpreted with some caution. As the targeted therapies are tested on different patient categories, with varying cancer genotypes, it is difficult to compare the presented results and draw conclusions on the efficacy of the investigated drugs. On the same note, the definition of the criteria for what is considered aberrant *MET* expression varies between the included trials, with some research groups applying IHC, some performing NGS, and some using different methods. A strength of this review is the variation in location of the included trials, leading to more generalizable and widely applicable conclusions. The exclusion criterion of a publication year prior to 2013 might be considered a limitation; however, this also ensured that only current results were included in this systematic review.

## 7. Conclusions

It remains difficult to compare different targeted therapies and to draw conclusions regarding their potential place in the future treatment panorama. The inter and intra study variation in cohort composition and included cancer genotypes is large. Onartuzumab has shown prolonged PFS and OS among *MET* positive NSCLC patients but no convincing results in cohorts with mixed *MET* mutational statuses. The studies on crizotinib lacked control groups for comparison of the outcome measures. Many ongoing trials on pipe-line targeted therapies exist, which are investigating anti-*MET* agents on clearly defined cohorts of patients with aberrant *MET* expression. This systematic review summarized the current status of publications on the *MET* gene’s implications in lung cancer development and the status of existing and up and coming targeted therapy options. More research is needed and should be encouraged to fully understand how, when, and to whom these drugs should be recommended in order to improve patient outcomes.

## Figures and Tables

**Table 1:** Overview of included *EGFR* and *MET* TKIs. *EGFR* and *MET* targeted therapies included in this systematic review. *EGFR*: epidermal growth factor receptor, *MET*: mesenchymal epithelial transition factor, TKI: tyrosine kinase receptor, *VEGFR2*: vascular endothelial growth factor receptor 2, *RET*: ret proto-oncogene, *ROS1*: ROS proto-oncogene 1, *KIT*: CD117, *TIE-2*: tyrosine kinase with immunoglobin and EGF homology domains 2, *AXL*: AXL receptor tyrosine kinase, RTK: receptor tyrosine kinase, NSCLC: non-small cell lung cancer, HGF: hepatocyte growth factor, ATP: adenosine triphosphate, CYP2C19: cytochrome P450 2C19.

Drug Name	Effect	References
Afatinib	Binds covalently and irreversibly to the kinase domain of *EGFR*.	Arrieta et al. [1]
Cabozantinib	A small molecule TKI that targets *MET*, *VEGFR2*, *RET*, *ROS1*, *KIT*, *TIE-2*, and *AXL*. Binds intracellularly to *MET*.	Neal et al. [2] Landi et al. [18]
Capmatinib	A highly selective intracellular *MET* inhibitor.	Schuler et al. [20] Sequist et al. [22]
Crizotinib	An intracellular *MET*/*ALK*/*ROS1* RTK inhibitor with high specificity for *MET*.	Landi et al. [18]
Dacomitinib	A small irreversible pan-human *EGFR* inhibitor.	Jänne et al. [21]
Erlotinib	A reversible, small-molecule *EGFR* TKI.	Spigel et al. [6]
Gefitinib	A reversible *EGFR* TKI.	Arrieta et al. [1] Wu et al. [23]
Onartuzumab	A recombinant, fully humanized, one-armed anti-*MET* monovalent monoclonal antibody. Binds to the extracellular domain of *MET* without activating it and without dimerizing.	Hirsch et al. [17] Kishi et al. [15]
Osimertinib	A CNS-active, irreversible *EGFR* TKI.	Sequist et al. [22]
Rociletinib	An irreversible *EGFR* TKI targeting mutated form of the *EGFR* gene.	Arrieta et al. [1]
Savolitinib	A small molecule, ATP competitive, selective *MET* TKI.	Sequist et al. [22]
Tepotinib	A highly selective, ATP competitive *MET* inhibitor.	Wu et al. [23]
Tivantinib	A selective, non-ATP-competitive *MET* inhibitor metabolized by CYP2C19.	Yoshioka et al. [5]

**Table 2:** Characteristics of included articles reporting the prevalence and impact of *MET* and *HGF* expression. Summary of characteristics of the six included articles reporting numbers regarding the prevalence of aberrant *MET* expression and results regarding the impact of the protein HGF on outcome. NSCLC: non-small cell lung cancer, EGFR: epidermal growth factor receptor, WT: wild type, NS: mutational status not specified, TKI: tyrosine kinase inhibitor, HGF: hepatocyte growth factor, ORR: objective response rate, PFS: progression-free survival, OS: overall survival, MET: mesenchymal epithelial transition factor, GCN: gene copy number, METex14: MET exon 14 skipping mutations.

	Total Participants	Cancer Type	Mutational Status	Active Drug	Results
Arrieta et al., 2016 [1]	*n* = 66	NSCLC	*EGFR* WT *EGFR*+	Afatinib (*EGFR* TKI)	Reduced levels of HGF led to improved ORR, PFS, and OS.
Helman et al., 2018 [25]	*n* = 77	NSCLC	*EGFR*+	Rociletinib (*EGFR* TKI)	Prevalence of *MET* alteration was 15.0%. *MET* amplification was seen in 7.6% of these, 4.5% had focal amplification, and 3.1% had aneuploidy.
Matsumoto et al., 2014 [19]	*n* = 47	NSCLC	*EGFR* WT	Erlotinib (*EGFR* TKI)	Expression of HGF resulted in poor response to erlotinib with shorter PFS. *MET* mutational status did not correlate to response to erlotinib or PFS.
Okamoto et al., 2014 [3]	*n* = 295	NSCLC	NS	Chemotherapy	Totally, 21 patients (7.6%) had *MET* mutations. *MET* amplifications were present in 9 (3.9%) cases. Median GCN was 8.8 among *MET* amplified patients.
Palmero et al., 2021 [11]	*n* = 186	NSCLC	NS	None	Totally, 22.0% of patients had *MET* amplifications and 11.0% had *MET*ex14 mutations.
Sacher et al., 2016 [28]	*n* = 22	NSCLC	*EGFR* WT *EGFR*+	Erlotinib	Totally, 45.0% of subjects harbored a *MET* alteration. *MET* amplification was present in 9.0% of the patients.

**Table 3:** Characteristics of included articles reporting effect of targeted therapies. Summary of characteristics and results of the 16 included articles that reported the effect of specific targeted therapies. NSCLC: non-small cell lung cancer, EGFR: epidermal growth factor receptor, WT: wild type, MET: mesenchymal epithelial transition factor, NS: mutational status not specified, IHC: immunohistochemistry, GCN: gene copy number, CEP7: centromere 7, METex14: MET exon 14 skipping mutations, NGS: next generation sequencing, TKI: tyrosine kinase inhibitor, VEGF-A: vascular endothelial growth factor A, PFS: progression-free survival, OS: overall survival, ns: not significant, ORR: objective response rate, 2/3L: second- or third-line.

	Study Design	Total Participants	Cancer Type	Mutational Status	Definition of *MET*+	Active Drug	Results
Han et al., 2017 [14]	Phase III	*n* = 636	NSCLC	*EGFR*+/WT, *MET*+/−	Not defined	Onartuzumab (*MET* TKI) + erlotinib (*EGFR* TKI) vs. erlotinib	High dose onartuzumab resulted in longer PFS (4.4 months) compared to low dose (2.5 months) and erlotinib (2.5 months). No significant difference in OS.
Hirsch et al., 2017 [17]	Phase II	*n* = 106	NSCLC	*MET*+/−	*MET* IHC2+ or IHC3+	Onartuzumab (*MET* TKI) + chemotherapy vs. chemotherapy + placebo	Median PFS was 5 months for onartuzumab and 5.2 months for placebo (ns). Median OS was 10.8 months for onartuzumab and 7.9 months for placebo (ns).
Jänne et al., 2016 [21]	Phase I	*n* = 67	NSCLC	*EGFR*+/WT	*MET* IHC2+ or IHC3+, *MET* GCN ≥ 2.1	Crizotinib (*MET* TKI) + dacomitinib (*EGFR* TKI)	Median PFS was 3 months with 61.0% stable disease in the escalation phase. Median PFS was 2.1 months with 32.0% stable disease in the expansion phase.
Kishi et al., 2019 [15]	Phase II	*n* = 61	NSCLC	*EGFR*+ *MET*+	*MET* IHC2+ or IHC3+, the total number of *MET* genes in 20 cancer cells ≥ 90	Onartuzumab (*MET* TKI) + erlotinib (*EGFR* TKI)	Median PFS was 8.5 months, median OS 15.6 months, and ORR 68.9%.
Landi et al., 2019 [18]	Phase II	*n* = 26	NSCLC	*EGFR* WT *MET*+	*MET*-CEP7/ratio ≥ 2.2, *MET*ex14 mutation	Crizotinib (*MET* TKI)	ORR of 27.0%, median PFS 4.4 months, and median OS 5.4 months.
Moro-Sibilot et al., 2019 [29]	Phase II	*n* = 53	NSCLC	*EGFR* WT *EGFR*+ *MET*+/−	*MET* IHC2+ or IHC3+, *MET* GCN ≥ 6, *MET* exon skipping mutations in exon 14, 16–19 determined by NGS	Crizotinib (*MET* TKI)	ORR of 16.0%, median PFS of 3.2 months, and median OS of 7.7 months in the *MET* GCN ≥ 6 cohort. ORR of 10.7%, median PFS of 2.4 months, and median OS of 8.1 months in the *MET* mutated cohort.
Neal et al., 2016 [2]	Phase II	*n* = 111	NSCLC	*EGFR* WT	Tested through IHC, positive if *MET* was expressed in either membrane or cytoplasm	Cabozantinib (*MET* TKI) + erlotinib (*EGFR* TKI) vs. cabozantinib vs. erlotinib	Median PFS of 1.8 months and OS of 5.1 months for erlotinib. PFS was 4.7 months and OS was 12.3 months for erlotinib + cabozantinib. PFS was 4.3 months and OS was 9.2 months (ns) for cabozantinib. No association between *MET* IHC+ and PFS in the cabozantinib group.
Ou et al., 2017 [24]	Phase I	*n* = 26	NSCLC	NS	Mutational status not mentioned	Crizotinib (*MET* TKI) + erlotinib (*EGFR* TKI)	Two patients had partial response, 8 had stable disease, and 10 had progressive disease.
Scagliotti et al., 2018 [30]	Phase III	*n* = 109	NSCLC	*EGFR*+ *MET*+/−	*MET* IHC2+ or IHC3+, *MET* GCN ≥ 4	Tivantinib (*MET* TKI) + erlotinib (*EGFR* TKI) vs. erlotinib + placebo	Greater overall response rate (60.7%) and median PFS (13.0 months) for tivantinib + erlotinib compared to erlotinib + placebo (43.4%, 7.5 months). Similar median OS between groups (25.5 months for tivantinib and 20.3 months for placebo).
Schuler et al., 2020 [20]	Phase I	*n* = 55	NSCLC	*EGFR* WT *MET*+	*MET* H-score ≥ 150, *MET*/CEP7 ≥ 2.0, *MET* GCN ≥ 5, *MET* IHC 2+ or IHC3+	Capmatinib (*MET* TKI)	Median PFS was 3.7 months. In patients with *MET* GCN ≥ 6 median PFS was 9.3 months.
Sequist et al., 2020 [22]	Phase Ib	*n* = 180	NSCLC	*EGFR*+ *MET*+	*MET* GCN ≥ 5, *MET*/CEP7 ratio ≥ 2, *MET* IHC3+ or ≥20.0% tumor cells in NGS	Savolitinib (*MET* TKI) + osimertinib (*EGFR* TKI)	Partial response in 89 patients treated with savolitinib and osimertinib. PFS was 7.6 months and 9.1 months in two different subgroups.
Seto et al., 2021 [31]	Phase II	*n* = 45	NSCLC	*EGFR* WT *MET*+	*MET*ex14 mutation, *MET* amplification	Capmatinib (*MET* TKI)	In a subcohort with *MET*ex14 mutations receiving second or third-line (2/3L) treatment with capmatinib, overall response rate was 36.4%. In a second cohort with *MET* GCN ≥ 10, overall response rate to 2/3L capmatinib was 45.5%.
Spigel et al., 2013 [6]	Phase II	*n* = 136	NSCLC	*EGFR*+/WT *MET*+/−	*MET* IHC2+ or IHC3+	Onartuzumab (*MET* TKI) + erlotinib (*EGFR* TKI) vs. erlotinib	No difference in PFS and OS between groups. In a *MET*+ subgroup, PFS was significantly longer for onartuzumab compared to erlotinib + placebo (2.9 vs. 1.5 months). Longer OS in the *MET*+ subgroup receiving onartuzumab (12.6 vs. 3.8 months).
Wakelee et al., 2017 [32]	Phase II	*n* = 259	NSCLC	*EGFR*+/WT *MET*+/−	*MET* IHC2+ or ICH3+	Cohort 1: onartuzumab (*MET* TKI) + bevacizumab (VEGF-A TKI) + chemotherapy vs. placebo + bevacizumab + chemotherapy. Cohort 2: onartuzumab + chemotherapy vs. placebo + chemotherapy	Cohort 1: longer median PFS on placebo (6.8 months) compared to onartuzumab (5.0 months). A *MET*+ subgroup had a median PFS of 4.8 months and OS of 9.9 months on onartuzumab vs. 6.9 months and 16.5 months on placebo. Cohort 2: median PFS of 4.9 months and median OS of 8.5 months on onartuzumab vs. 5.1 months and 13.7 months on placebo. In a *MET*+ subgroup, median PFS was 5.0 months and OS was 8.0 months on onartuzumab vs. 5.0 months and 7.6 months on placebo.
Wu et al., 2020 [23]	Phase Ib/II	*n* = 55	NSCLC	*EGFR*+ *MET*+	*MET* IHC2+ or IHC3+, *MET* GCN ≥ 5	Tepotinib (*MET* TKI) + gefitinib (*EGFR* TKI) vs. chemotherapy	Significantly longer OS (37.3 months vs. 13.1 months) and PFS (16.6 months vs. 4.2 months) for tepotinib + gefitinib in patients with *MET* IHC3+ or *MET* GCN ≥ 5.
Yoshioka et al., 2015 [5]	Phase III	*n* = 303	NSCLC	*EGFR* WT *MET*+/−	IHC with moderate/strong intensity ≥ 50.0% of tumor cells, *MET* GCN ≥ 4	Tivantinib (*MET* TKI) + erlotinib (*EGFR* TKI) vs. erlotinib + placebo	Significantly longer PFS for tivantinib + erlotinib (2.9 months) compared to erlotinib + placebo (2 months). No effect on OS.

**Table 4:** Ongoing clinical trials. Overview of currently ongoing clinical trials investigating potential pipe-line targeted therapies for the treatment of lung cancer with aberrant *MET* expression. NSCLC: non-small cell lung cancer, MET: mesenchymal epithelial transition factor, METex14: MET exon 14 skipping mutations, RTK: receptor tyrosine kinase, ATP: adenosine triphosphate, EGFR: epidermal growth factor receptor.

Clinical Trial ID	Study Design	Study Type	Total Participants	Cancer Type	Mutational Status	Active Drug and Effect
NCT02544633	Phase II	Non-randomized	*n* = 68	NSCLC	*MET* activating mutation *MET* amplification	MGCD265: oral RTK inhibitor targeting *MET*
NCT02920996	Phase II	Single arm	*n* = 12	NSCLC	*MET*ex14 mutation	Merestinib: reversible type II ATP-competitive *MET* inhibitor
NCT02896231	Phase I	Dose escalation	*n* = 37	NSCLC	*MET*+	PLB1001: selective *MET* inhibitor
NCT04270591	Phase Ib/II	Single arm	*n* = 183	NSCLC	*MET*ex14 mutation *MET* amplification *MET* overexpression	Glumetinib: selective *MET* inhibitor
NCT02648724	Phase I/II	Non-randomized	*n* = 57	NSCLC	*MET* amplification *MET*ex14 deletion	Sym015: monoclonal antibody mixture targeting *MET*
NCT03539536	Phase II	Single arm	*n* = 275	NSCLC	*MET*+	Telisotuzumab vedotin: antibody-drug conjugate targeting *MET*
NCT02609776	Phase I	Non-randomized	*n* = 780	Advanced NSCLC	Varying	Amivantamab: human bispecific antibody targeting *EGFR* and *MET*
NCT04077099	Phase I/II	Single arm	*n* = 82	NSCLC	Any *MET* alteration	REGN5093: human bispecific antibody targeting *MET*, inducing internalization and degradation

## Appendix A

### Search Queries

**Search query 1 PubMed (*n* = 183):**

(((lung cancer[MeSH Terms]) OR (lung cancer)) AND ((((((“met”[All Fields]) OR (“mesenchymal epithelial transition factor”[All Fields])) OR (hgfr)) OR (hepatocyte growth factor receptor)) OR (hgf receptor[MeSH Terms])) OR (hepatocyte growth factor receptor[MeSH Terms]))) AND (((((“prognosis”[All Fields]) OR (“mortality”[All Fields])) OR (“morbidity”[All Fields])) OR (“diagnosis”[All Fields])) OR (morbidity[MeSH Terms])) AND (clinicaltrial[Filter] OR randomizedcontrolledtrial[Filter])

**Search query 2 PubMed (*n* = 157):**

((((lung cancer[MeSH Terms]) OR (lung cancer)) AND ((((((“met”[All Fields]) OR (“mesenchymal epithelial transition factor”[All Fields])) OR (hgfr)) OR (hepatocyte growth factor receptor)) OR (hgf receptor[MeSH Terms])) OR (hepatocyte growth factor receptor[MeSH Terms]))) AND ((((((targeted therapy) OR (cancer therapy)) OR (targeted treatment)) OR (molecular targeted therapies[MeSH Terms])) OR (drug targeting[MeSH Terms])) OR (cancer treatment protocol[MeSH Terms]))) AND (((((“prognosis”[All Fields]) OR (“mortality”[All Fields])) OR (“morbidity”[All Fields])) OR (“diagnosis”[All Fields])) OR (morbidity[MeSH Terms])) AND (clinicaltrial[Filter] OR randomizedcontrolledtrial[Filter])

**Search query 1 Embase (*n* = 384):**

(‘lung cancer’ OR ‘lung cancer’/exp) AND (‘met’/exp OR met OR ‘mesenchymal epithelial transition factor’ OR hgfr OR ‘scatter factor receptor’) AND (‘prognosis’/exp OR prognosis OR mortality OR morbidity OR diagnosis) AND ([controlled clinical trial]/lim OR [randomized controlled trial]/lim)

**Search query 2 Embase (*n* = 62):**

(‘lung cancer’ OR ‘lung cancer’/exp) AND (‘met’/exp OR met OR ‘mesenchymal epithelial transition factor’ OR hgfr OR ‘scatter factor receptor’) AND (‘prognosis’/exp OR prognosis OR mortality OR morbidity OR diagnosis) AND (‘molecularly targeted therapy’/exp OR ‘molecularly targeted therapy’ OR ‘cancer therapy’ OR ‘antineoplastic protocol’ OR ‘drug targeting’) AND ([controlled clinical trial]/lim OR [randomized controlled trial]/lim)

## Appendix B

**Table A1:** Full PRISMA 2020 Checklist [27]. N/A: not applicable.

Section and Topic	Item #	Checklist Item	Location Where Item Is Reported
**TITLE**			
Title	1	Identify the report as a systematic review.	Page 1
**ABSTRACT**			
Abstract	2	See the PRISMA 2020 for Abstracts checklist.	Page 1, paragraph 2
**INTRODUCTION**			
Rationale	3	Describe the rationale for the review in the context of existing knowledge.	Page 2, paragraph 2
Objectives	4	Provide an explicit statement of the objective(s) or question(s) the review addresses.	Page 4, paragraph 2
**METHODS**			
Eligibility criteria	5	Specify the inclusion and exclusion criteria for the review and how studies were grouped for the syntheses.	Page 5, paragraph 2
Information sources	6	Specify all databases, registers, websites, organisations, reference lists and other sources searched or consulted to identify studies. Specify the date when each source was last searched or consulted.	Page 5, paragraph 1
Search strategy	7	Present the full search strategies for all databases, registers and websites, including any filters and limits used.	Page 22
Selection process	8	Specify the methods used to decide whether a study met the inclusion criteria of the review, including how many reviewers screened each record and each report retrieved, whether they worked independently, and if applicable, details of automation tools used in the process.	Page 5, paragraph 2
Data collection process	9	Specify the methods used to collect data from reports, including how many reviewers collected data from each report, whether they worked independently, any processes for obtaining or confirming data from study investigators, and if applicable, details of automation tools used in the process.	Page 5, paragraph 2
Data items	10a	List and define all outcomes for which data were sought. Specify whether all results that were compatible with each outcome domain in each study were sought (e.g., for all measures, time points, analyses), and if not, the methods used to decide which results to collect.	Page 6, paragraph 1
	10b	List and define all other variables for which data were sought (e.g., participant and intervention characteristics, funding sources). Describe any assumptions made about any missing or unclear information.	Page 6, paragraph 1
Study risk of bias assessment	11	Specify the methods used to assess risk of bias in the included studies, including details of the tool(s) used, how many reviewers assessed each study and whether they worked independently, and if applicable, details of automation tools used in the process.	Page 11, paragraph 1
Effect measures	12	Specify for each outcome the effect measure(s) (e.g., risk ratio, mean difference) used in the synthesis or presentation of results.	N/A
Synthesis methods	13a	Describe the processes used to decide which studies were eligible for each synthesis (e.g., tabulating the study intervention characteristics and comparing against the planned groups for each synthesis (item #5)).	Page 8, Table 3
	13b	Describe any methods required to prepare the data for presentation or synthesis, such as handling of missing summary statistics, or data conversions.	N/A
	13c	Describe any methods used to tabulate or visually display results of individual studies and syntheses.	Page 8, Table 3
	13d	Describe any methods used to synthesize results and provide a rationale for the choice(s). If meta-analysis was performed, describe the model(s), method(s) to identify the presence and extent of statistical heterogeneity, and software package(s) used.	N/A
	13e	Describe any methods used to explore possible causes of heterogeneity among study results (e.g., subgroup analysis, meta-regression).	N/A
	13f	Describe any sensitivity analyses conducted to assess robustness of the synthesized results.	N/A
Reporting bias assessment	14	Describe any methods used to assess risk of bias due to missing results in a synthesis (arising from reporting biases).	Page 11, paragraph 1
Certainty assessment	15	Describe any methods used to assess certainty (or confidence) in the body of evidence for an outcome.	N/A
RESULTS			
Study selection	16a	Describe the results of the search and selection process, from the number of records identified in the search to the number of studies included in the review, ideally using a flow diagram.	Page 6, Figure 3
	16b	Cite studies that might appear to meet the inclusion criteria, but which were excluded, and explain why they were excluded.	N/A
Study characteristics	17	Cite each included study and present its characteristics.	Page 11, paragraph 4
Risk of bias in studies	18	Present assessments of risk of bias for each included study.	Pages 27–28
Results of individual studies	19	For all outcomes, present, for each study: (a) summary statistics for each group (where appropriate) and (b) an effect estimate and its precision (e.g., confidence/credible interval), ideally using structured tables or plots.	Pages 29–30
Results of syntheses	20a	For each synthesis, briefly summarise the characteristics and risk of bias among contributing studies.	Page 21, paragraph 2
	20b	Present results of all statistical syntheses conducted. If meta-analysis was done, present for each the summary estimate and its precision (e.g., confidence/credible interval) and measures of statistical heterogeneity. If comparing groups, describe the direction of the effect.	N/A
	20c	Present results of all investigations of possible causes of heterogeneity among study results.	Page 21, paragraph 2
	20d	Present results of all sensitivity analyses conducted to assess the robustness of the synthesized results.	N/A
Reporting biases	21	Present assessments of risk of bias due to missing results (arising from reporting biases) for each synthesis assessed.	Pages 27–28
Certainty of evidence	22	Present assessments of certainty (or confidence) in the body of evidence for each outcome assessed.	N/A
DISCUSSION			
Discussion	23a	Provide a general interpretation of the results in the context of other evidence.	Page 18, paragraph 1
	23b	Discuss any limitations of the evidence included in the review.	Page 21, paragraph 2
	23c	Discuss any limitations of the review processes used.	Page 21, paragraph 2
	23d	Discuss implications of the results for practice, policy, and future research.	Page 18, paragraph 3
OTHER INFORMATION			
Registration and protocol	24a	Provide registration information for the review, including register name and registration number, or state that the review was not registered.	Page 5, paragraph 1
	24b	Indicate where the review protocol can be accessed, or state that a protocol was not prepared.	Page 5, paragraph 1
	24c	Describe and explain any amendments to information provided at registration or in the protocol.	N/A
Support	25	Describe sources of financial or non-financial support for the review, and the role of the funders or sponsors in the review.	Page 22
Competing interests	26	Declare any competing interests of review authors.	Page 22
Availability of data, code and other materials	27	Report which of the following are publicly available and where they can be found: template data collection forms; data extracted from included studies; data used for all analyses; analytic code; any other materials used in the review.	Page 22

**Table A2:** PRISMA Abstract Checklist [27].

Topic	No.	Item	Reported?
**TITLE**			
Title	1	Identify the report as a systematic review.	Yes
**BACKGROUND**			
Objectives	2	Provide an explicit statement of the main objective(s) or question(s) the review addresses.	Yes
**METHODS**			
Eligibility criteria	3	Specify the inclusion and exclusion criteria for the review.	No
Information sources	4	Specify the information sources (e.g., databases, registers) used to identify studies and the date when each was last searched.	Yes
Risk of bias	5	Specify the methods used to assess risk of bias in the included studies.	No
Synthesis of results	6	Specify the methods used to present and synthesize results.	No
**RESULTS**			
Included studies	7	Give the total number of included studies and participants and summarise relevant characteristics of studies.	Yes
Synthesis of results	8	Present results for main outcomes, preferably indicating the number of included studies and participants for each. If meta-analysis was done, report the summary estimate and confidence/credible interval. If comparing groups, indicate the direction of the effect (i.e., which group is favoured).	Yes
**DISCUSSION**			
Limitations of evidence	9	Provide a brief summary of the limitations of the evidence included in the review (e.g., study risk of bias, inconsistency and imprecision).	No
Interpretation	10	Provide a general interpretation of the results and important implications.	Yes
**OTHER**			
Funding	11	Specify the primary source of funding for the review.	No
Registration	12	Provide the register name and registration number.	Yes

## Appendix C

**Table A3:** Epidemiology reported by the included studies. Median age, percentage of participants that are male gender, and smoking history, presented as percentage of participants with a history of no smoking, of the included patients in the different studies and their subgroups. EGFR: epidermal growth factor receptor, WT: wild type, MET: mesenchymal epithelial transition factor, NS: not specified.

	Mutational Status	Median Age (Years)	Male Gender (%)	History of No Smoking (%)
Arrieta et al., 2016 [1]	*EGFR*+/WT	60.1	33.3%	39.4%
Han et al., 2017 [14]	*EGFR*+/WT, *MET*+/−	Treatment: 63.0 Placebo: 63.0	Treatment: 56.0% Placebo: 57.0%	Treatment: 18.5% Placebo: 15.8%
Helman et al., 2018 [25]	*EGFR*+	61.0	28.6%	NS
Hirsch et al., 2017 [17]	*MET*+/−	Treatment: 68.0 Placebo: 66.0	Treatment: 70.9% Placebo: 74.1%	Treatment: 3.6% Placebo: 5.6%
Jänne et al., 2016 [21]	*EGFR*+/WT	59.5	39.0%	51.0%
Kishi et al., 2019 [15]	*EGFR*+ *MET*+	67.0	43%	NS
Landi et al., 2019 [18]	*EGFR* WT *MET*+	56.0	65%	23%
Matsumoto et al., 2014 [19]	*EGFR* WT	64.0	19.7%	89.1%
Moro-Sibilot et al., 2019 [29]	*EGFR*+/WT *MET*+/−	*MET* amplified: 59.0 *MET* mutated: 72.0	*MET* amplified: 56.0% *MET* mutated: 32.0%	*MET* amplified: 24.0% *MET* mutation: 48.0%
Neal et al., 2016 [2]	*EGFR* WT	65.3	45.0%	15%
Okamoto et al., 2014 [3]	NS	64.0	77.0%	18.0%
Ou et al., 2017 [24]	NS	60.0	30.0%	52.0%
Palmero et al., 2021 [11]	NS	64.3	65.0%	27.0%
Sacher et al., 2016 [28]	*EGFR*+/WT	64.0	45.0%	18.0%
Scagliotti et al., 2018 [30]	*EGFR*+ *MET*+/−	Treatment: 59.5 Placebo: 65.0	Treatment: 42.9% Placebo: 47.2%	Treatment: 48.2% Placebo: 60.4%
Schuler et al., 2020 [20]	*EGFR* WT *MET*+	60.0	60.0%	NS
Sequist et al., 2020 [22]	*EGFR*+ *MET*+	Cohort B: 59.0 Cohort D: 62.0	Cohort B: 59% Cohort D: 60%	NS
Seto et al., 2021 [31]	*EGFR* WT *MET*+	68.0	66.7%	42.2%
Spigel et al., 2013 [6]	*EGFR*+/WT *MET*+/−	Treatment *MET*-: 63.0 Placebo *MET*-: 61.0 Treatment *MET*+: 66.0 Placebo *MET*+: 64.0	Treatment *MET*-: 65.0% Placebo *MET*-: 55.0% Treatment *MET*+: 51.0% Placebo *MET*+: 65.0%	Treatment *MET* -: 7.0% Placebo *MET* -: 3.0% Treatment *MET*+: 20.0% Placebo *MET*+: 19.0%
Wakelee et al., 2017 [32]	*EGFR*+/WT *MET*+/−	Cohort I treatment: 60.0 Cohort I placebo: 60.5 Cohort II treatment: 66.0 Cohort II placebo: 63.0	Cohort I treatment: 68.1% Cohort I placebo: 48.6% Cohort II treatment: 55.9% Cohort II placebo: 42.6%	Cohort I treatment: 21.7% Cohort I placebo: 25.7% Cohort II treatment: 27.1% Cohort II placebo: 13.1%
Wu et al., 2020 [23]	*EGFR*+ *MET*+	Phase Ib: 65.5 Phase II treatment: 61.0 Phase II placebo: 58.3	Phase Ib: 44.0% Phase II treatment: 35.5% Phase II placebo: 50.0%	Phase Ib: 72.0% Phase II treatment: 68.0% Phase II placebo: 67.0%
Yoshioka et al., 2015 [5]	*EGFR* WT *MET*+/−	Treatment: 63.0 Placebo: 63.0	Treatment: 66.7% Placebo: 70.8%	Treatment: 25.5% Placebo: 26.2%
